# Longitudinal follow-up of muscle echotexture in infants with congenital muscular torticollis

**DOI:** 10.1097/MD.0000000000006068

**Published:** 2017-02-10

**Authors:** Ching-Fang Hu, Tieh-Cheng Fu, Chung-Yao Chen, Carl Pai-Chu Chen, Yu-Ju Lin, Chih-Chin Hsu

**Affiliations:** a Department of Physical Medicine and Rehabilitation, Keelung Chang Gung Memorial Hospital, Keelung; b Department of Physical Medicine and Rehabilitation, Chang Gung Memorial Hospital, Linkou; c School of Medicine; d School of Traditional Chinese Medicine, College of Medicine, Chang Gung University, Taoyuan, Taiwan.

**Keywords:** fibrosis, infant, torticollis, ultrasonography

## Abstract

Unilateral fibrous contracture of the sternocleidomastoid (SCM) muscle is the major pathophysiology in infants with congenital muscular torticollis (CMT). Physical examination is not always sufficient to detect minimal muscle fibrosis in involved SCM muscles.

A prospective study for SCM muscle fibrosis in CMT infants by quantifying echotexture and muscle thickness during the course of treatment is highlighted in the study.

Convenience samples of 21 female and 29 male infants with CMT, who were 1 to 12 months old, underwent physiotherapy for at least 3 months and were followed for 4.7 ± 0.4 months. All infants had at least 2 clinical assessments and ultrasonographic examinations for bilateral SCM muscles during follow-up. The *K* value, derived from the difference in echo intensities between the involved and uninvolved SCM muscles on longitudinal sonograms, was used to represent the severity of muscle fibrosis. Bilateral SCM muscle thickness and ratio of involved to uninvolved muscle thickness (Ratio I/U) were obtained simultaneously. Clinical outcome was also recorded.

No subjects underwent surgical intervention during follow-up. The *K* value decreased from 6.85 ± 0.58 to 1.30 ± 0.36 at the end of follow-up (*P* < 0.001), which reflected the decrease of muscle fibrosis. The Ratio I/U decreased from 1.11 ± 0.04 to 0.97 ± 0.02 during treatment, which was possibly related to the increased uninvolved SCM muscle thickness.

In conclusion, echotexture is an efficient indicator for reflecting a wide degree of muscle fibrosis in infants with CMT and is informative during the treatment course.

## Abstract 2

Supplemental Digital Content is available in the text

## 1 Introduction

Infants with congenital muscular torticollis (CMT) feature unilateral fibrous contracture of the sternocleidomastoid (SCM) muscle with a characteristic head tilt, limited neck rotation, or a palpable mass.^[1]^ Physical examination is not always sufficient in diagnosing CMT in infants with minimal clinical presentations, and image workup is sometimes required for determining the treatment strategy.^[1,2]^ Because of lack of related cohort studies, randomized controlled trials, or meta-analyses, imaging has been suggested as level II evidence for infants with CMT, but clinical practice guidelines still recommend that physical therapists obtain all image interpretations before informing prognosis.^[2]^ Compression sonoelastography^[3,4]^ and color Duplex images^[5]^ have been used to help describe the degree of SCM muscle fibrosis. However, lacking constant loading force and quantification of blood flow in target muscles, these new technologies represent less objective analyses for SCM muscle fibrosis.

Ultrasonography (US) can be used for quantifying the degree of muscle fibrosis,^[6]^ defining the size and location of muscle masses,^[1]^ and guiding the clinical approach and treatment duration in CMT.^[1,7]^ This imaging type has been widely advocated because of the ability to identify different patterns of SCM muscle fibrosis.^[1,2,7,8]^ Most infants showed a pseudotumor (type I fibrosis) or diffuse fibrosis mixed with normal muscle and thickening of involved muscles (type II fibrosis).^[1]^ The muscle fibrosis pattern was altered during follow-up, and a certain of them turned into total fibrosis (type III fibrosis) or fibrous bands (type IV fibrosis), with surgical intervention indicated.^[1,7]^ A duplicate blinded examination of different examiners has been done to assess the interobserver variation for the above US classifications and κ value was 1.00 in the interrater agreement.^[9]^ Although there is high concordance in the US classification, we still believe that only digitalized sonograms can totally eradicate not only inter- but also intraobserver errors.

US can accurately measure SCM muscle thickness in CMT infants, and revealed a significant reduction in ratio of involved to uninvolved SCM muscle thickness (Ratio I/U) after adequate rehabilitation.^[10]^ Findings in muscle thickness measurement may reflect clinical improvement but cannot directly reflect the progress of muscle fibrosis during follow-up. Therefore computer-assisted analysis has been developed to quantify the muscle echo intensity (EI) to directly associate the muscle sonogram findings with the severity of muscle fibrosis.^[6,11]^

Echotextures on sonograms are the result of reflected waves from interfaces between materials of different acoustic impedance and reflect different muscle pathologies.^[1,6,12]^ The skeletal muscle consists of grouped muscle fibers separated by fibroadipose septa.^[13]^ This tissue arrangement results in inhomogeneous and speckled muscle sonograms.^[14]^ Strong correlations were observed between percentage of intramuscular fat seen on magnetic resonance imaging and muscle EI.^[15]^ Quantification of muscle EI has also been found objective and reproducible and is used in screening dystrophic and inflammatory myopathies.^[15]^ Animal studies showed muscle EI highly correlated with the extent of fibrosis in affected muscles^[6,16]^ and US characterization was found useful in estimating muscle fibrosis.^[6]^ However, the application of muscle EI in clinical surveys of nondystrophic muscle disorders is still limited.

We wished to quantify the therapeutic effect of physiotherapy directly through the tissue characterization of involved muscles instead of indirectly through the clinical evaluation. The *K* value, derived from the difference in mean echo intensity (*MEI*) between the involved and uninvolved muscle in every examination under the same ultrasound setting, reflects the severity of fibrosis in affected muscles.^[6]^ The present work aimed to document echotexture in CMT infants by the *K* value and thickness of bilateral SCM muscles derived from US during the treatment course. Findings concerning echotexture and muscle thickness alterations during follow-up may provide additional insights into the progression of SCM muscle fibrosis with physiotherapy.

## 2 Methods

### 2.1 Participants

The institutional review board of a tertiary care hospital approved the study protocol (100-4436B) and the clinical trial registry number is NCT02889705. CMT infants whose parents gave signed informed consent were prospectively enrolled. Infants with neurological, cervical spine abnormalities, or developmental dysplastic hip problems were excluded. The presenting clinical features, including head tilt in the upright position, facial asymmetry, limited passive range of motion in neck rotation, palpable neck mass, and results of US were carefully evaluated and documented at every visit. Age, gender distribution, body weight (BW), body length (BL), characteristics of the affected muscle side, follow-up duration, visit times, and interval between 2 visits were also recorded. Physiotherapy involving passive stretching of the involved muscle, positioning, and massage can diminish the development of scoliosis and facial asymmetry for CMT infants.^[1,7,17]^ All infants received the above physiotherapy 2 to 4 times a week for at least 3 months and were regularly followed at our rehabilitation clinic. Those who still had prominent clinical presentations after physiotherapy for 6 months or were older than 1 year would undergo surgery. Presenting clinical features that subsided with physiotherapy were determined by the clinician.

### 2.2 Procedures

**Figure 1:**
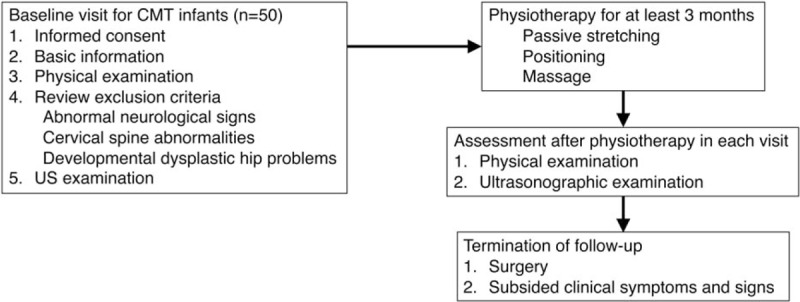
Flowchart of infant selection, intervention, and follow-up.

The study was a prospective, experimental pre–post design with 1 participant group. Figure 1 illustrates the selection of infants and follow-up.

### 2.3 Quantification of US findings

An experienced sonographer performed US examinations with the infant in the supine position and the head rotated contralaterally to the examination side. A 5 to 12 MHz linear-array ultrasound transducer (Philips iU22, Philips Healthcare, Andover, MA) was used to obtain longitudinal and transverse views of bilateral SCM muscles. The ultrasound system settings, including gain (86%), monitor dynamic range (70 dB), and depth (2 cm), were kept constant throughout the study.

**Figure 2:**
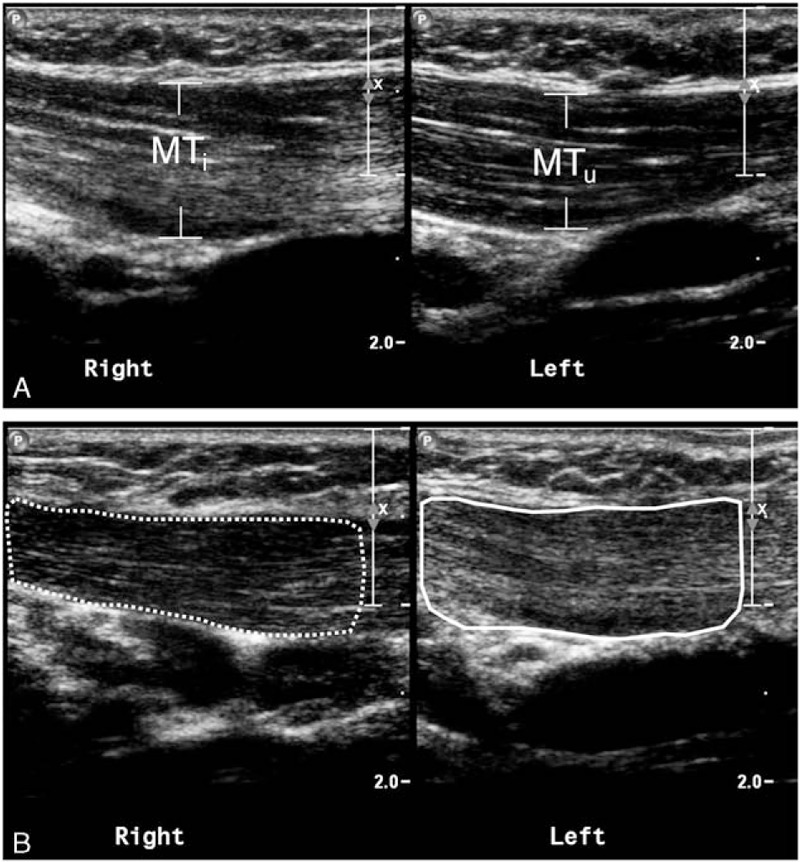
US of bilateral SCM muscles in infants with CMT. (A) Longitudinal sonograms of maximum anterior–posterior diameters of the involved (MT_i_) and uninvolved (MT_u_) SCM muscles. Ratio I/U was calculated as MT_i_/MT_u_. (B) Longitudinal sonograms of the *MEI* of every pixel in the ROI in the uninvolved (dotted line) and involved (solid line) muscles. The *K* value was calculated by *MEI*_uninvolved_ − *MEI*_involved_.

All infants underwent at least 2 US measurements and 35 of them had ≥3 examinations (6 had ≥4 examinations, and 1 of the 6 had 7 examinations). Sonograms for involved and uninvolved muscles were compared. The maximum anterior–posterior diameter, defined as muscle thickness, was measured from the longitudinal view, then the Ratio I/U was derived (Fig. 2A). The muscle EI was determined by computer-assisted gray-scale analysis and calculated using MATLAB 2006b (The MathWorks, Natick, MA). The *MEI* of every pixel in the region of interest (ROI) and the *K* value used to compare sonograms between infants or between times for the same infant were estimated by the following equations (Fig. 2B)^[6]^: 
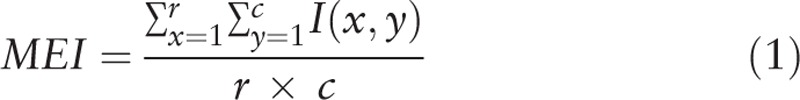
 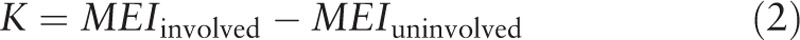


where *r* and *c* represent the pixel counts for the height and width of the ROI on the sonogram, respectively. A decrease in *K* value indicates reduced muscle fibrosis.^[6]^

### 2.4 Statistical analysis

All data are presented as mean ± standard error of mean. Because only 1 infant underwent US more than 4 times, the 5th to 7th measurements for this infant were incorporated into the 4th measurement for statistical analysis. Initial US images in 10 different subjects were analyzed as the above procedure in the same program on the same International Business Machines Corporation-compatible personal computer by 2 different operators. Each obtained image was analyzed 10 times and the coefficient of variance ranged from 3% to 5% in each examination. The analyzed values of each image between different operators were similar. A generalized estimating equation was used to compare different US measurements of muscle thickness (involved and uninvolved SCM muscles) and the *K* value during follow-up. Student *t* test was used to compare the thickness between bilateral SCM muscles. Pearson correlation was used to analyze the relationship between US measurements, especially the initial examination, and clinical information. *P* < 0.05 was considered statistically significant.

## 3 Results

### 3.1 Clinical information

We prospectively recruited 50 infants for the study (21 females and 29 males) with the mean age of 4.3 ± 0.3 months (range 1–12). Among them, 23 infants had left and 27 had right SCM muscle involvement. Their initial mean BW and BL of all subjects was 7.0 ± 0.2 kg and 63.4 ± 0.9 cm, respectively, and was about 10 kg and about 80 cm at the end of follow-up. The mean follow-up period and interval were 4.7 ± 0.4 months (range 1.4–14.7) and 2.5 ± 0.2 months (range 0.7–12.6), respectively. All included infants had facial asymmetry and limited neck rotation to the lesion side. Four had a palpable neck mass (right/left: 3/1). The clinical symptoms and signs subsided after physiotherapy of different durations. Therefore, no CMT infants had surgical intervention at the end of follow-up.

### 3.2 Ultrasonographic measurement of echotexture

**Figure 3:**
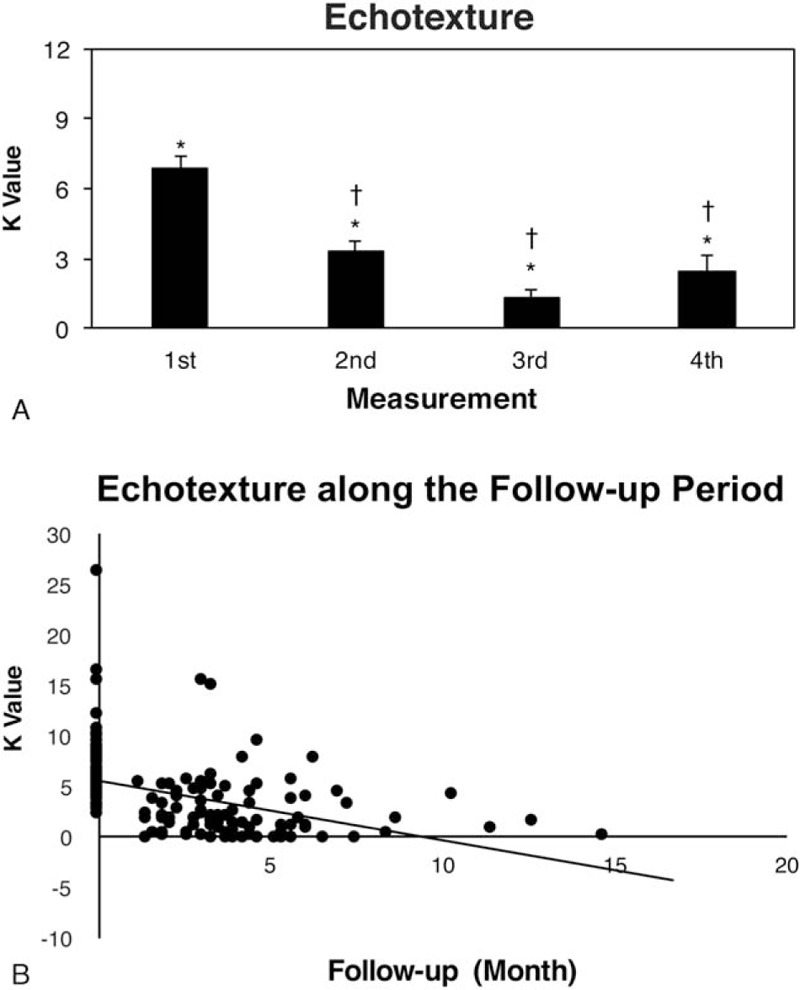
Measurements of (A) *K* value and (B) scatter diagrams for *K* value during follow-up. (A) ∗*P* values between the 1st and 2nd, 3rd, and 4th measurements (all *P* < 0.001). ^†^*P* values between the 2nd and 3rd (*P* < 0.001) and 4th measurements (*P* = 0.015). (B) The *K* value of involved SCM muscles shows a negative-slope linear regression trend line (solid line).

Each CMT infant underwent US examination for a mean of 2.9 ± 1.2 times (range: 2–7) during follow-up. The initial mean *K* value for all CMT infants was 6.85 ± 0.58 (range 2.22–26.23) and decreased to 1.30 ± 0.36 (range 0–7.72) during follow-up (*P* < 0.001) (Fig. 3A). The high 4th measurement for 6 CMT infants was possibly related to the severe SCM muscle fibrosis as reflected in the high mean initial *K* value of 11.3 ± 3.1 (range 6.06–26.23) for these infants. One infant, showing severe fibrosis by a high initial *K* value of 12.1, underwent US examination of 7 times and had a high *K* value at the 5th measurement (Supplementary Fig. 1).

We found a linear regression trend line with a negative slope of −0.02 for *K* value during follow-up (Fig. 3B). This finding represented decreased echotexture difference between involved and uninvolved SCM muscles during follow-up.

### 3.3 Ultrasonographic measurement of muscle thickness

**Figure 4:**
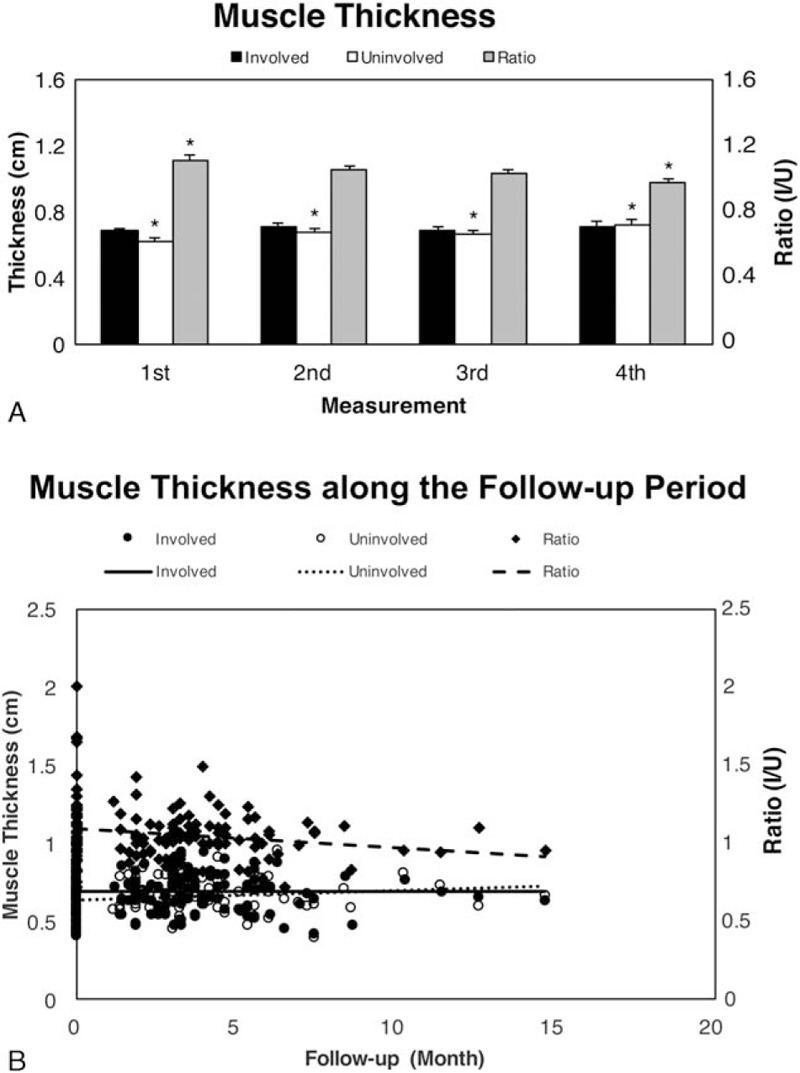
Measurement of (A) muscle thickness and (B) scatter diagrams for muscle thickness during follow-up. (A) Difference in involved muscle thickness (black bar) between each measurement was not significant. ∗*P* values for the uninvolved muscles (white bar) between the 1st and 2nd (*P* = 0.004), 3rd (*P* = 0.039), and 4th measurements (*P* = 0.026). ∗*P* value for Ratio I/U between the 1st and 4th measurement (*P* = 0.006). (B) Linear regression trend lines for uninvolved (dotted line) and involved (solid line) SCM muscle thickness, and for Ratio I/U (dashed line) during follow-up.

Involved and uninvolved SCM muscle thickness ranged from 0.6 to 0.8 cm during follow-up. The differences between involved and uninvolved SCM muscle thickness at each US measurement were not significant. The differences in involved SCM muscle thickness during follow-up also were not significant. However, we observed significant differences in uninvolved SCM muscles between measurements 1 (0.62 ± 0.02 cm) and 2 (0.68 ± 0.02 cm, *P* = 0.004), 3 (0.67 ± 0.02 cm, *P* = 0.039), and 4 (0.69 ± 0.07 cm, *P* = 0.026). The initial mean Ratio I/U was 1.11 ± 0.04 (range: 0.78–1.48). This ratio decreased progressively during follow-up to the 0.97 ± 0.02 (range: 0.83–1.10), with a significant difference between the 1st and 4th measurement (*P* = 0.006) (Fig. 4A).

A mildly increased trend of uninvolved and nearly no change in involved SCM muscle thickness occurred during follow-up (Fig. 4B). We also observed a linear regression trend line for Ratio I/U during follow-up, with a negative slope of −0.0004. This observation was possibly related to the increase in uninvolved SCM muscle thickness over time.

### 3.4 Correlation between the ultrasonographic measurement and clinical information

The *K* value, reflecting the degree of muscle fibrosis, was negatively and fairly correlated with age, follow-up duration, and number of US examinations. *K* value was negatively and weakly correlated with BW and BL, which suggested the effects of growth and development on muscle fibrosis. *K* value was positively and weakly correlated with involved SCM muscle thickness and Ratio I/U. The initial *K* value was positively correlated with follow-up duration, number of US examination, and involved muscle thickness. Fibrotic SCM muscle thickness was fairly correlated with normal SCM muscle thickness and Ratio I/U. Uninvolved SCM muscle thickness was poorly correlated with number of US examinations and negatively with Ratio I/U. Detailed information is listed in Table 1.

**Table 1:**
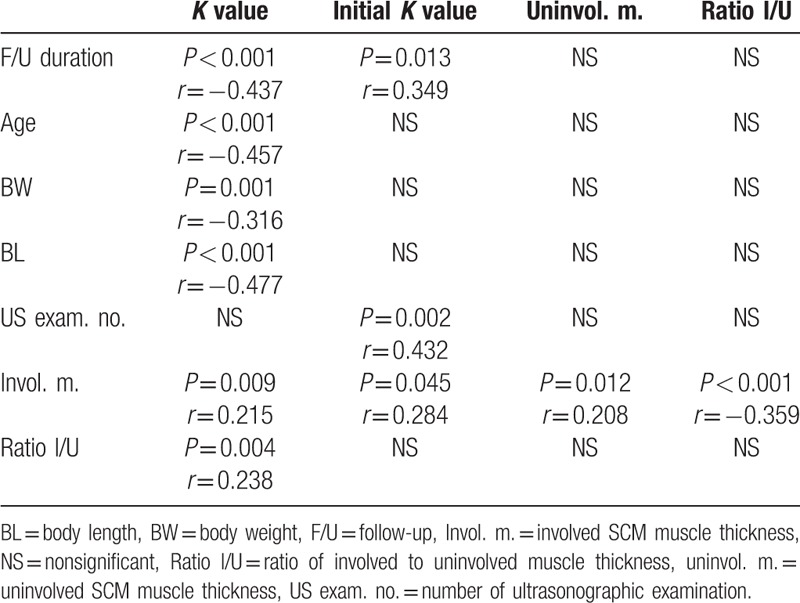
Correlation between ultrasonographic measurement and clinical information.

## 4 Discussion

US examination is helpful in daily practice because it is noninvasive and reflects underlying pathological changes without the need for muscle biopsy and patient cooperation. In a typical muscle fibrosis image, the normal muscle architecture is disrupted by infiltrated collagen fibers, which cause increased reflection of the ultrasound beam and results in increased muscle EI.^[1,6,12,16,18]^ Varied US system settings during each examination and operators with different experience are responsible for the high intra- and interobserver errors during US interpretation based on muscle EI.^[18,19]^ Therefore, the *K* value was developed to decrease study error and increase experimental reliability.^[6]^ The initial *K* values of all our infants with CMT varied from 2.22 to 26.23, which represented a wide degree of fibrosis in affected SCM muscles rather than only 2 different types of fibrosis. After receiving regular physiotherapy, our CMT infants showed a significant decrease in *K* values during follow-up on serial sonograms of affected SCM muscles (Fig. 3A). The decrease in *K* value indicates that the MEI for involved muscle approaches that for uninvolved muscle, for overall decreased muscle fibrosis.^[6]^

Muscle biopsy specimens from patients with neuromuscular disease show common histopathologic findings of atrophied muscle fibers and increased perimysial fibrosis.^[20]^ Muscle EI has been clinically used to quantify severity of muscle fibrosis in patients with Duchenne muscular dystrophy (DMD),^[21]^ inflammatory myopathy,^[12]^ metabolic myopathy,^[22]^ and lumbar radiculopathy.^[23]^ The muscle EI of serial muscle images was used in the follow-up of boys 4 to 12 years old with DMD and was inversely related to ambulatory status, functional grading, muscle strength, and motor ability.^[21]^ A study used the Heckmatt scale to follow the spasticity of involved gastrocnemius muscles in stroke patients before and after botulinum toxin-A injection.^[24]^ The EI is difficult to differentiate in different muscle pathologies, but it seemed a good candidate for reflecting the clinical status during the course of treatment from the above reports.

Besides the muscle fibrosis, growth and development may affect muscle components.^[25,26]^ The uninvolved muscle thickness increased along the follow-up period representing muscle growth effects in the study. Thickness changes of involved muscles, reflecting the complex of decreased fibrosis after physiotherapy and increased normal muscle component with growth, were not distinctive. Selective activation of protein kinase B caused by the release of insulin growth factor-1 from muscle fibers during passive stretch promotes myosatellite cell proliferation and induces skeletal muscle hypertrophy.^[27,28]^ Stretch-induced antifibrotic effects, as much as the well-known antifibrotic agent decorin, has also been reported in injured gastrocnemius muscles of rats.^[29]^ Therefore, both normal growth and development and physiotherapy may affect the improvement of SCM muscle fibrosis as reflected in the change in *K* value during follow-up. The *K* value is useful for continuously observing severity of SCM muscle fibrosis and may be a competent indicator in mirroring the progression of muscle fibrosis during treatment.

With pure imaging, sonograms can tell us only the change in fibrosis type or sustained decrease in muscle fibrosis in the involved SCM muscle during the treatment course for CMT infants. However, the image presentation lacks a digitized indicator to show the amelioration of fibrosis in CMT infants after physiotherapy, which can be demonstrated by the *K* value (Supplementary Fig. 1). In contrast, changes in sequential measurements of the involved SCM muscle thickness and differences between bilateral SCM muscles during the treatment course were not significant. We found an increasing trend of uninvolved muscle thickness during follow-up, with a significant increase at about 3 months (2.9 ± 0.2 months) (Fig. 4A). Uninvolved SCM muscle thickness in all infants slowly increased with follow-up duration (Fig. 4B). Ratio I/U tended to decreased during treatment, with no decrease in involved SCM muscle thickness during follow-up (Fig. 4B). The significant negative correlation between uninvolved muscle thickness and Ratio I/U was largely due to the increase in uninvolved SCM muscle thickness.

As compared with changes in muscle thickness and thickness ratio during treatment, the improvement in echotexture was noticeable. Nevertheless, our results showed that involved SCM muscle thickness was not related to treatment period. Both these findings suggest that the *K* value is a better candidate than muscle thickness in describing the therapeutic effect on affected SCM muscles during physiotherapy. From our observations, infants with high initial *K* value, implying severe fibrosis, will receive a long treatment course and frequent US examinations. More frequent US examinations and longer physiotherapy course may be required for infants with greater degree of SCM muscle fibrosis.

### 4.1 Study limitations

US is a valuable workup tool for following the clinical status of CMT infants but cannot elucidate the pathogenesis of the muscle fibrosis. Echotexture analysis and thickness measurement of involved SCM muscles are end-clinical presentations of CMT infants. Study design only correlated the *K* value with the existence of clinical symptoms of head tilting, facial asymmetry, and neck range of motion, instead of the severity of clinical presentation. We will stress on this field in the near future. Muscle histopathological results were not available, because no subjects in the study underwent surgical intervention. Therefore, it is hard to differentiate the effects of maturation, therapy, or growth on involved muscle in the study.

## 5 Conclusions

In addition to decreasing fibrosis, both growth and development and stretch-induced muscle hypertrophy affect the muscle architecture in affected SCM muscles in infants with CMT. The muscle EI reflects muscle tissue characterization and is correlated with muscle fibrosis in different disease entities.^[12,21–23]^ The *K* value, derived from the difference between the involved and uninvolved muscle *MEI*, can aid in following the progression of muscle fibrosis in CMT infants during treatment. Quantification of muscle fibrosis is a digitized and sensitive indicator reflecting a wide degree of SCM muscle fibrosis in CMT and can be of great help in clinical practice.

## Supplementary Material

Supplemental Digital Content
