# Supplementary material for: Longitudinal follow-up of muscle echotexture in infants with congenital muscular torticollis
Source: Medicine (Baltimore). 2017 Feb 10;96(6):e6068. doi: 10.1097/MD.0000000000006068 (PMC5313018; doi:10.1097/MD.0000000000006068)

**Supplementary Figure 1**: Sequential echotexture, muscle thickness measurement, and US images in a 1-month-old female CMT infant during a follow-up of 441 days. A: The K value increased to 15.4 in the second measurement possibly because of the change in morphology from type I to II fibrosis. Thereafter, the value decreased progressively to 0.04 at the end of follow-up. B: The involved and uninvolved SCM muscle thickness was 0.95 and 0.57 cm. The bilateral muscle thickness resolved gradually and a similar thickness was observed after 6 months of physiotherapy. Thereafter, bilateral muscle thickness was maintained at a similar level. A similar trend was observed in Ratio I/U. C: A pseudotumor (cursors), type I fibrosis, in the left SCM muscle observed at Day 0. Diffuse fibrosis mixed with normal muscles (type II fibrosis) in the left SCM muscle identified about 3 months after initial evaluation. Serial images show a progressive alteration in gray-scale images in left SCM muscle and thickness changes in bilateral SCM muscles.


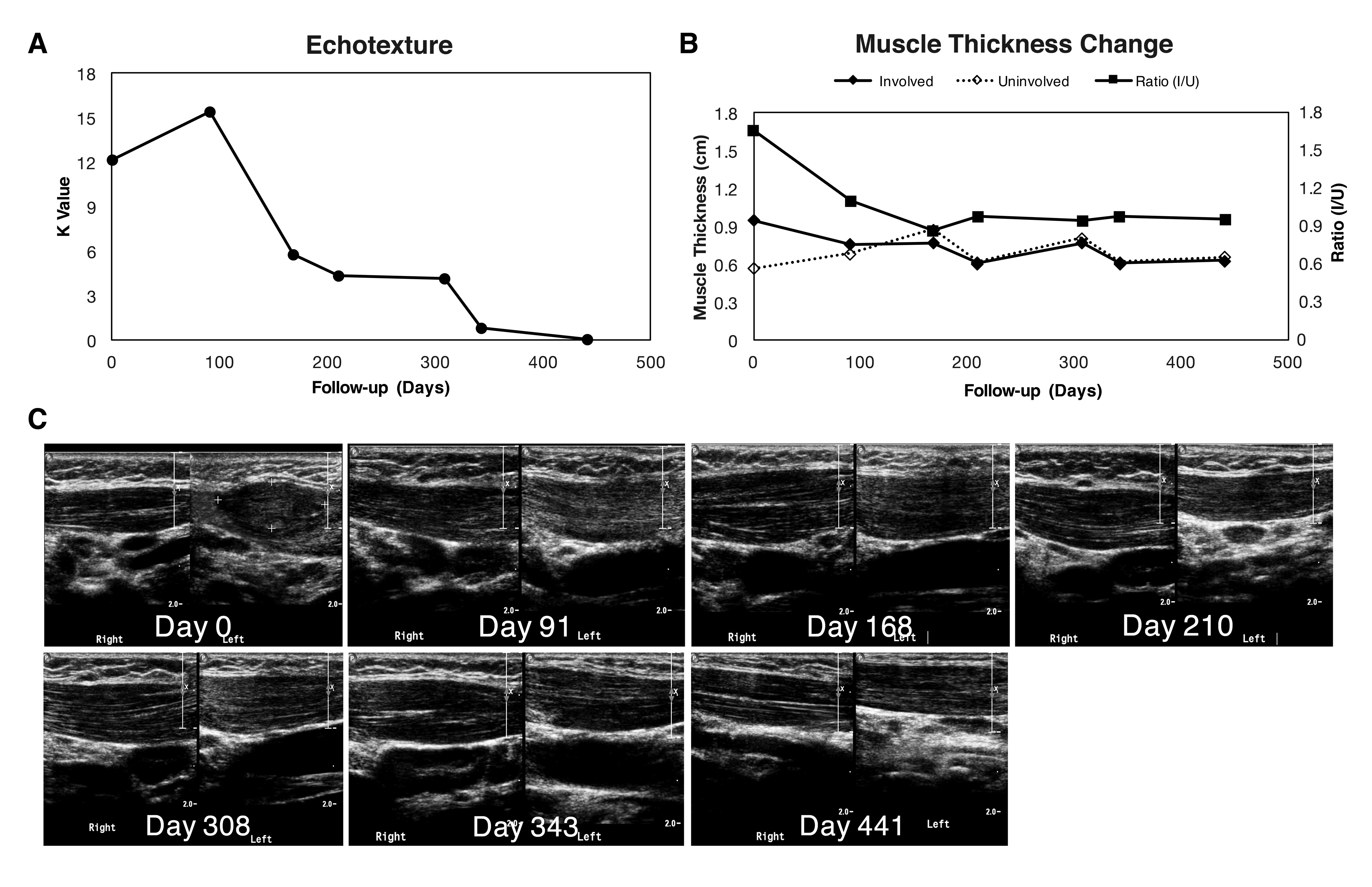

Supplement: Supplemental Digital Content [file medi-96-e6068-s001.docx]
